# Developing a novel reference region for [^18^F]PI-2620-PET imaging to facilitate the assessment of 4-repeat tauopathies

**DOI:** 10.1007/s00259-025-07396-8

**Published:** 2025-06-10

**Authors:** Lukas Frontzkowski, Johannes Gnörich, Mattes Gross, Amir Dehsarvi, Sebastian N. Roemer-Cassiano, Carla Palleis, Sabrina Katzdobler, Anna Dewenter, Anna Steward, Davina Biel, Fabian Hirsch, Zeyu Zhu, Johannes Levin, Andrew W. Stephens, Andre Müller, Norman Koglin, Gérard N. Bischof, Gabor G. Kovacs, Günter U. Höglinger, Matthias Brendel, Nicolai Franzmeier

**Affiliations:** 1 Institute for Stroke and Dementia Research (ISD), University Hospital, LMU Munich, Feodor-Lynen-Straße 17, Munich, 81377 Germany; 2 Department of Nuclear Medicine, LMU University Hospital, Marchioninistraße 15, Munich, 81377 Germany; 3 Department of Neurology, LMU University Hospital, Munich, Germany; 4 Munich Cluster for Systems Neurology (SyNergy), Munich, Germany; 5 German Center for Neurodegenerative Diseases (DZNE), Munich, Germany; 6 Life Molecular Imaging, GmbH, Berlin, Germany; 7 Tanz Centre for Research in Neurodegenerative Disease (CRND), Toronto, Canada; 8 Laboratory Medicine Program and Krembil Brain Institute, University Health Network, Toronto, Canada; 9 The Sahlgrenska Academy, Institute of Neuroscience and Physiology, Department of Psychiatry and Neurochemistry, University of Gothenburg, Mölndal and Gothenburg, Sweden

**Keywords:** Tau, [^18^F]PI-2620, Progressive supranuclear palsy, Four-repeat tauopathies, Reference region

## Abstract

**Purpose:**

Progressive supranuclear palsy (PSP) is a fatal 4-repeat (4R) tauopathy with progressive movement phenotypes. In-vivo 4R tau biomarkers are therefore crucial for PSP diagnosis, monitoring, and treatment evaluation. The tau-PET tracer [^18^F]PI-2620 binds to 4R tau and shows increased uptake in PSP-associated regions (e.g., globus pallidus), and is therefore a candidate 4R tau biomarker. However, commonly used cerebellar tau-PET reference regions show regional proximity to cerebellar 4R tau deposits in PSP, confounding semiquantitative [^18^F]PI-2620 assessments. Therefore, we employed bias-free image-derived input function (IDIF) PET quantification to identify an optimized data-driven reference region for assessing 4R tau in PSP.

**Methods:**

Dynamic [^18^F]PI-2620 PET (60 min) was acquired in 58 PSP-Richardson Syndrome (PSP-RS) and 18 healthy controls (HC). IDIF-modelling with carotid timeseries derived total distribution volume (VT). Iteratively normalizing VT images to atlas-based white matter (WM), we identified reference candidates maximizing PSP-RS vs. HC pallidum differences. The best-performing WM references were combined to a temporo-orbital WM reference, validated in PSP-nonRS (*n* = 54), HC (*n* = 18), and disease controls (α-synucleinopathies, *n* = 21; Alzheimer’s disease (AD, *n* = 22) using VT-ratios (VTr) and 20-40min static standardized uptake value ratios (SUVr).

**Results:**

Using the data-driven temporo-orbital WM reference, PSP patients showed significantly higher basal ganglia [^18^F]PI-2620 signal vs. HC compared to cerebellar normalization. Receiver operating curve (ROC) analysis confirmed higher diagnostic accuracy using the temporo-orbital WM reference. Pallidum [^18^F]PI-2620 showed significant associations with clinical disease severity exclusively when using the novel temporo-orbital WM reference.

**Conclusions:**

A data-driven temporo-orbital WM reference optimizes [^18^F]PI-2620 PET assessment for PSP diagnosis, outperforming conventional cerebellar references used in tau-PET imaging.

**Supplementary Information:**

The online version contains supplementary material available at 10.1007/s00259-025-07396-8.

## Introduction

Neurodegenerative 4-repeat (4R) tauopathies are characterized by cerebral 4R tau accumulation, leading to progressive motor and cognitive dysfunction [1–4]. The most common manifestations of 4R tauopathies are progressive supranuclear palsy (PSP) with Richardson syndrome (PSP-RS) and cortico-basal syndrome (CBS), both of which share clinical similarities with the α-synucleinopathy Parkinson’s disease (PD) or the secondary 3/4R tauopathy atypical Alzheimer’s disease (AD) [5, 6]. Currently, the diagnosis of PSP is based purely on clinical criteria, with definitive confirmation only post-mortem, as no sensitive and specific in vivo biomarkers are clinically established, which hinders molecularly defined in vivo identification of PSP pathology [7–10]. Recent advances in tau-PET imaging have drastically improved the detection of tau in the secondary 3/4R tauopathy AD, which has transformed biomarker-based diagnosis, staging, prognosis and treatment monitoring in AD [11, 12]. However, 1 st generation tau tracers have failed to identify PSP-type 4R pathology in vivo due to off-target binding in typical 4R aggregation sites such as the pallidum and overall low affinity to 4R tau [13–16]. In contrast, the 2nd generation tau-PET tracer [^18^F]PI-2620 binds to PSP-type 4R and AD-type 3/4R tau in vitro [17–19] and shows elevated [^18^F]PI-2620 PET binding in PSP in subcortical 4R predilection sites such as the pallidum and putamen [15, 20–24]. Further, autoradiography studies illustrated [^18^F]PI-2620 co-localization with 4R tau deposits in post-mortem PSP tissue [15, 20, 25], which we further confirmed by showing [^18^F]PI-2620 PET to autopsy correlations of tau severity in PSP patients [25]. Conclusively, these findings render [^18^F]PI-2620 a promising candidate to detect 4R tau in vivo in PSP [26–28]. Therefore, establishing [^18^F]PI-2620 as a 4R tau biomarker holds the potential to transform molecularly informed PSP diagnosis, to gain mechanistic insights into PSP pathophysiology, to benchmark other candidate 4R tau biomarkers and to track disease progression in clinical trials.

However, optimal quantification of [^18^F]PI-2620 PET remains challenging due to low to moderate increases in tracer uptake in typical 4R tau-vulnerable subcortical regions compared to the 3/4R tauopathy AD, where cortical tau-PET increases are much stronger [15, 29]. We argue that a potential key confound of [^18^F]PI-2620 PET quantification in 4R tauopathies like PSP is the choice of an optimal reference region used to scale the tau-PET signal in pathology harboring regions against an ideally pathology free region. The common standard for tau-PET assessment is intensity normalization to an inferior cerebellar grey matter (GM) reference, which has been developed for assessment of tau pathology in AD, where the cerebellum typically shows little to no tau pathology [30, 31]. This is, however, problematic as post-mortem studies found that 4R tau accumulates in the cerebellar dentate nucleus and in oligodendrocytes in close vicinity to the cerebellar cortex in PSP, thereby potentially confounding PET quantification when using a cerebellar GM reference [24, 32, 33]. In addition, part of the cerebellar vermis and falx cerebri are typical off-target binding sites for [^18^F]PI-2620 due to binding to (neuro)melanin [34, 35]. Therefore, it is crucial to determine a more suitable reference region for optimal quantification of 4R tau, to enhance [^18^F]PI-2620 PET usage for assessing PSP in clinical and research settings. Hence, our major goal was to improve [^18^F]PI-2620 PET quantification in 4R tauopathies by identifying an optimized reference region for 4R tau, using a fully data-driven approach.

**Fig. 1:**
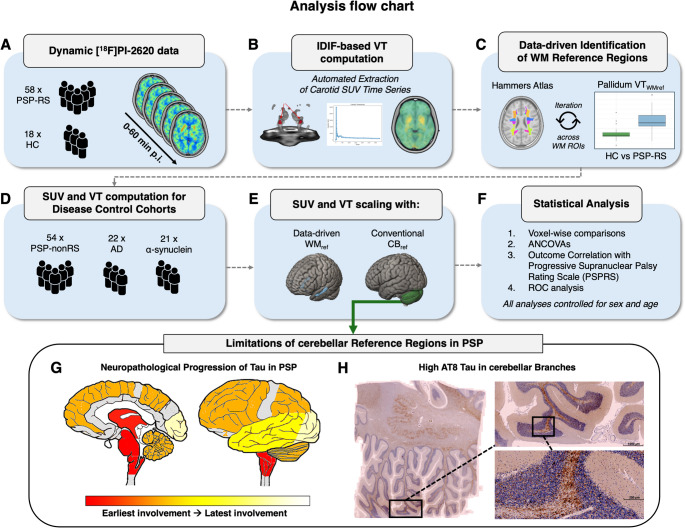
Analysis flow chart. **A** Dynamic 0–60 min [^18^F]PI-2620 PET data of 58 patients with PSP-RS and 18 HC were used to compute **B** IDIF-based VT images by an automated extraction method of carotid SUV timeseries. **C** Using a data-driven approach, 27 bilateral WM regions obtained from the Hammers Atlas were iteratively tested as potential candidates for intensity-normalization. Emerging WM regions leading to the largest group differences between PSP-RS patients and HC were combined into one new reference region spanning the temporo-orbital WM (WM_ref_). **D** Using 20–40 min SUVr and VTr from PSP-RS, PSP-nonRS, AD, α-synuclein and HC, **E** both references were compared by **F** voxel-wise comparisons, ANCOVAs, association with PSP rating scale and ROC analysis. Limitations of Cerebellar Reference Regions in PSP. **G** Neuropathological progression of tau in PSP as evidenced by Kovacs et al. showcasing that 4R tau is accumulating in oligodendrocytes that are located in WM branches in the vicinity to cerebellar GM in later disease stages [24]. **H** AT8 immunohistochemistry results of cerebellar PSP tissue showing the accumulation of tau in vicinity to GM

To this end, we used dynamic 0–60 min [^18^F]PI-2620 tau-PET from a discovery cohort of 58 PSP-RS patients and 18 healthy controls (HC). We quantified cerebral [^18^F]PI-2620 PET via an unbiased image-derived input function (IDIF) approach, using a pre-established automated extraction of carotid PET timeseries to calculate total volume of distribution (VT) images without the usage of a pre-defined tissue reference [36]. We have shown previously that carotid-based IDIF quantification of [^18^F]PI-2620 PET is strongly correlated to invasive arterial sampling, therefore offering a non-invasive alternative for reference region free quantification of cerebral PET tracer uptake [36]. IDIF-based VT images were iteratively referenced to atlas-based bilateral white-matter (WM) regions to identify an optimized PET reference region that allows optimal PSP-RS vs. HC group discrimination using the pallidum as a pre-established visual and quantitative 4R targeting readout [15, 37]. WM reference candidate regions leading to the strongest PSP vs. HC group differences were merged into one novel reference. This data-driven reference region was then applied to an independent validation cohort of PSP-nonRS (*n* = 54) and disease controls (AD = 22, α-synuclein = 21) to compute intensity-normalized IDIF-based VT ratios (VTr) as well as 20–40 min standard uptake value ratios (SUVr) for evaluating its’ use in pre-established PSP-tailored static imaging protocols for [^18^F]PI-2620 PET [37, 38]. Using these data, we evaluated the novel data-driven WM reference region against the conventional inferior cerebellar GM reference by comparing mean SUVr‘s and VTr’s derived from typical 4R (i.e., pallidum) and 3/4 accumulation sites (i.e. temporal meta region) between PSP, HC, AD and α-synuclein patients for cross-disease comparisons. Using receiver operating curve (ROC) analysis, we further determined the diagnostic performance of [^18^F]PI-2620 PET referenced to either temporo-orbital WM or inferior cerebellar GM and determined cut-off values for identifying PSP. Lastly, we investigated whether higher pallidum [^18^F]PI-2620 PET predicted stronger PSP-associated functional deficits. An overview of our overall approach is depicted in Fig. 1.

## Methods

### Sample

We included 58 patients with PSP-RS as well as 54 PSP–nonRS patients as a validation sample. All PSP-RS and -nonRS patients were amyloid-biomarker negative based on routine CSF or amyloid-PET assessments to rule out concomitant AD pathology. The PSP-nonRS sample consisted of individuals with CBS (*n* = 41), PSP with predominant frontal presentation (PSP-F, *n* = 6), predominant parkinsonism (PSP-P, *n* = 4), predominant speech/language disorder (PSP-SL, *n* = 1), predominant progressive gait freezing (PSP-PGF, *n* = 1) and predominant primary lateral sclerosis (PSP-PLS, *n* = 1). Further, we included 43 disease controls, i.e., 21 patients with α-synucleinopathies (α-syn) and 22 amyloid-biomarker positive (i.e., CSF or PET) patients across the AD spectrum. The α-syn sample consisted of individuals with dementia with Lewy bodies (DLB, *n* = 4), Parkinson’s disease (PD, *n* = 13), Parkinson’s disease with dementia (PDD, *n* = 1) as well as multiple system atrophy of the cerebellar type (MSA-C, *n* = 2) and Parkinsonian type (MSA-P, *n* = 1) [7, 39, 40]. As a HC reference sample, we included 18 subjects without any evidence of neurological or psychiatric disorders. In a subset of PSP patients, we further employed the PSP rating scale (PSPRS) as a marker of PSP-typical clinical disease severity [41]. Written informed consent for PET-imaging was collected from all participants, and ethical approval was obtained by the institutional ethics committee at the University Hospital of Munich, LMU Munich, Germany (application numbers 17–569, 19–022).

### Dynamic [^18^F]PI-2620 PET imaging

[^18^F]PI-2620 was synthesized as previously described [38]. PET imaging was performed at the Department of Nuclear Medicine at the LMU University Hospital in a full dynamic setting (0–60 min post-injection (p.i.)) using a Siemens Biograph True point 64 PET/CT or Siemens mCT (Siemens, Erlangen, Germany). The administered activity dose ranged between 156 and 218 MBq (median administered activity: 189 MBq) applied as a slow (10 s) intravenous bolus injection. The dynamic brain PET data was acquired in list-mode over 60 min and reconstructed into 35 time frames (12 × 5 s, 6 × 10 s, 3 × 20 s, 7 × 60 s, 4 × 300 s and 3 × 600 s) using a 336 × 336 × 109 matrix (voxel size: 1.02 × 1.02 × 2.0 mm3) and the built-in 3-dimensional ordered subset expectation maximization (OSEM) algorithm with 4 iterations, 21 subsets and a 5 mm full-width-at-half-maximum Gaussian filter on the Siemens Biograph and with 5 iterations, 24 subsets and a 5 mm full-width-at-half-maximum Gaussian filter on the Siemens mCT. A CT was used for attenuation correction (tube voltage: 120 kV, tube current: 33 mA, pitch: 1.5, rotation time: 0.5 s). As scatter correction, single scatter simulation was used.

### Image processing and automated image derived input function modelling of dynamic [^18^F]PI-2620 PET data

VT images were computed using a previously established in-house dynamic PET pipeline, which computes an IDIF using an automated extraction of carotid artery SUV timeseries [36]. First, dynamic [^18^F]PI-2620 PET images were motion corrected via a stepwise co-registration using FSL, subsequently averaged into a mean 20–40 min PET image which was warped to the Montreal Neurology Institute (MNI) space via a 20–40 min [^18^F]PI-2620 custom in-house MNI summation template using the advanced normalization tools (ANTs) software. To compute IDIF, an independent component analysis (ICA) with a pre-defined 10 component solution was applied to the native space dynamic PET image, parcellating the image with the rationale that voxels belonging to the carotid artery show a highly temporally correlated SUV signal during early frames of the dynamic scan. The resulting component maps were warped to MNI space using the prior obtained ANTs-derived high-dimensional warping parameters, spatially correlated with a custom in-house carotid artery template to select subject-specific carotid components maps and then masked using a binary image which restricts the carotid artery to a segment in the upper part of the pars cervicalis. Using the ANTs derived warping parameters, the masked subject-specific carotid image was warped back to PET native space with nearest-neighbor interpolation to preserve a binary image and further eroded using FSL to eliminate signal confounding voxels close to the vessel walls. Finally, the eroded binary carotid image was adjusted to the native space dynamic PET image to extract the SUV timeseries across the 60-minute [^18^F]PI-2620 PET scan [38]. IDIFs were generated by automated extraction of the maximum PET signal intensity from the carotid mask over the 60-minute dynamic PET scan, using our pre-established approach that closely resembles timeseries determined via an invasive arterial input function [36]. For quantification of [^18^F]PI-2620 PET binding differences, VT images were calculated using Logan plots [42] and spatially normalized to MNI space, using the ANTs derived normalization parameters.

### Data-driven identification of a [^18^F]PI-2620 reference region for 4R tauopathies

**Fig. 2:**
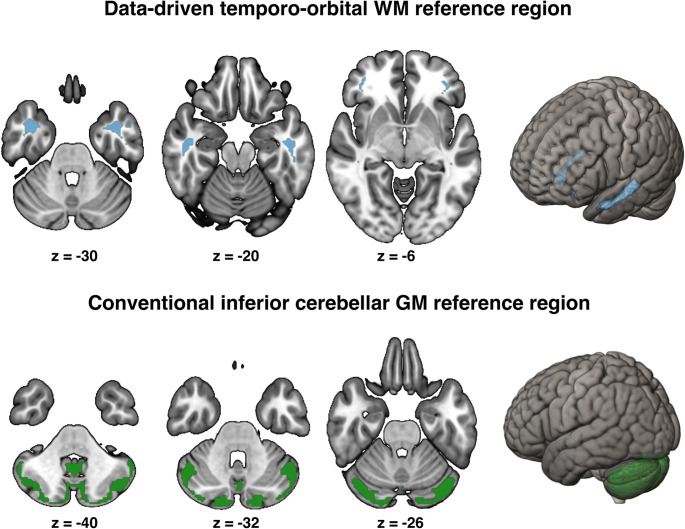
Reference region visualization. Structural MRI slices and brain renderings were used to visualize temporo-orbital WM (first row) and inferior cerebellar GM (second row)

Through an iterative approach, IDIF-based VT images were intensity-normalized (i.e. VTr) to 27 bilateral WM regions of the Hammers Brain Atlas [43] to identify reference regions where pallidum VTr values show the largest PSP-RS vs. HC group difference. The pallidum was selected as key readout as it resembles a typical 4R predilection site in both PSP-RS and PSP-nonRS as determined on post-mortem and [^18^F]PI-2620 tau-PET data [4, 15, 24, 44]. All tested WM regions were first eroded using FSL and a 2 mm kernel to minimize potential spillover of confounding PET signal from adjacent grey matter structures [45]. Subcortical nuclear or cortical structures were excluded from the analyses as these regions have a higher likely-hood of specific or non-specific tau-tracer uptake and are thus not suitable as reference for intensity-normalization. Using those VTr images defined for each WM ROI of the Hammers atlas, we determined PSP-RS vs. HC group differences in pallidum VTr’s, using ANCOVAs adjusted for age and sex. Subsequently, WM references surviving an FWE-based multiple comparison correction (*p* < 0.05) were combined into a single reference spanning the bilateral temporal and orbital WM (Fig. 2, Supp. Table 1). For subsequent in-depth comparison of this novel data-driven temporo-orbital WM reference vs. the conventional inferior cerebellar GM reference, we determined VTr and static 20–40-minute [^18^F]PI-2620 PET SUVRs for PSP-RS, PSP-nonRS (*n* = 54), disease controls (i.e., α-syn, *n* = 21; AD, *n* = 22) and HC (*n* = 18) adjusting to temporo-orbital WM and inferior cerebellar GM, respectively (Fig. 2, Supp. Figs. 1 and 2).

**Table 1:** Sample characteristics

Variable	PSP-RS (*n *= 58)	PSP–nonRS (*n *= 54)	AD (*n *= 22)	α-syn (*n *= 21)	HC (*n *= 18)	*P*
Subgroups	PSP-RS	PSP-CBS (*n* = 41)	NA	PD (*n *= 13)	NA	
		PSP-F (*n* = 6)		DLB (*n *= 4)		
		PSP-P (*n *= 4)		PDD (*n *= 1)		
		PSP-SL (*n* = 2)		MSA-C (*n *= 2)		
		PSP-PLS (*n *= 1)		MSA-P (*n *= 1)		
Age	70.6±7.9	71.7±6.3	73.8±9.8	63.0±10.5	71.7±8.4	0.0001
Sex (m/f)	35/23	30/24	12/10	15/6	9/9	0.664
PSPRS*	34.1±9.2	29.1±14.0	NA	NA	NA	0.0618

Age and PSPRS values are presented as mean±SD. *PSPRS scores were available for a subset of 40 PSP-RS patients and 40 PSP-nonRS patients

### Statistics

MATLAB version R2023b (Statistical Parameter Mapping Software, The MathWorks Inc., Portola Valley, United States) and R Version 4.4.1 were used for statistical testing [46]. All analyses were conducted for intensity scaled VT (i.e. VTr) and SUV (i.e., SUVr) data, that were intensity normalized to a conventional inferior cerebellar GM or the data-driven temporo-orbital WM reference. To assess binding differences in SUVr and VTr PET data between PSP and disease samples with HC, a cluster-corrected voxel-wise comparison (voxel-level *p* < 0.001, cluster level p_FWE_<0.05) was conducted using SPM12 (Wellcome Trust Centre for Neuroimaging, University College London), adjusting for age and sex. Further, a ROC analysis was performed to estimate the optimal [^18^F]PI-2620 SUVr and VTr thresholds for discrimination of PSP-RS and PSP-nonRS groups from HC. Areas under the curve (AUC) for both reference regions were compared using a non-parametric approach as previously described [47]. For targeted across-disease group assessments, analysis of covariance (ANCOVA) adjusted for age and sex was employed to test for differences between average [^18^F]PI-2620 SUVr and VTr values in the pallidum and the AD-tailored temporal meta ROI [48] between all cohorts. Pairwise comparisons were conducted to correct for multiple testing using the Tukey adjustment, and effect sizes (Cohen’s d) were calculated for between group comparisons. To evaluate associations between mean SUVr and VTr pallidum scores and the PSPRS as a measure of clinical disease severity, linear regression models adjusted for age and sex were applied. P values less than 0.05 were considered significant (* *p* < 0.05, ** *p* < 0.01, *** *p* < 0.001).

## Results

### Sample description

Sample demographics and clinical characteristics are shown in Table 1. Overall, we included 58 PSP-RS patients (35 men [60.3%]; mean [SD] age, 70.6 [7.9] years; mean [SD] PSPRS score, 34.1 [9.2]) and 54 PSP-nonRS patients (30 men [55.6%]; mean [SD] age, 71.7 [6.3] years; mean [SD] PSPRS score, 29.1 [14.0]). PSPRS scores capturing clinical disease severity were available for a subset of patients (PSP-RS = 40, PSP-nonRS = 40) and did not differ between PSP-RS and non-RS (*p* = 0.06). In addition, we included 22 AD patients (12 men [54.5%]; mean [SD] age, 73.8 [9.8] years) and 21 α-syn patients (15 men [71.4%]; mean [SD] age, 63.0 [10.5] years) as disease controls as well as 18 HC (9 men [50%]; mean [SD] age, 71.7 [8.4] years). There was a significant difference in age between groups, where α-syn patients were younger than PSP and AD groups (*p* < 0.05).

### Validation of the data-driven temporo-orbital WM reference for assessment of 4R tauopathies

**Fig. 3:**
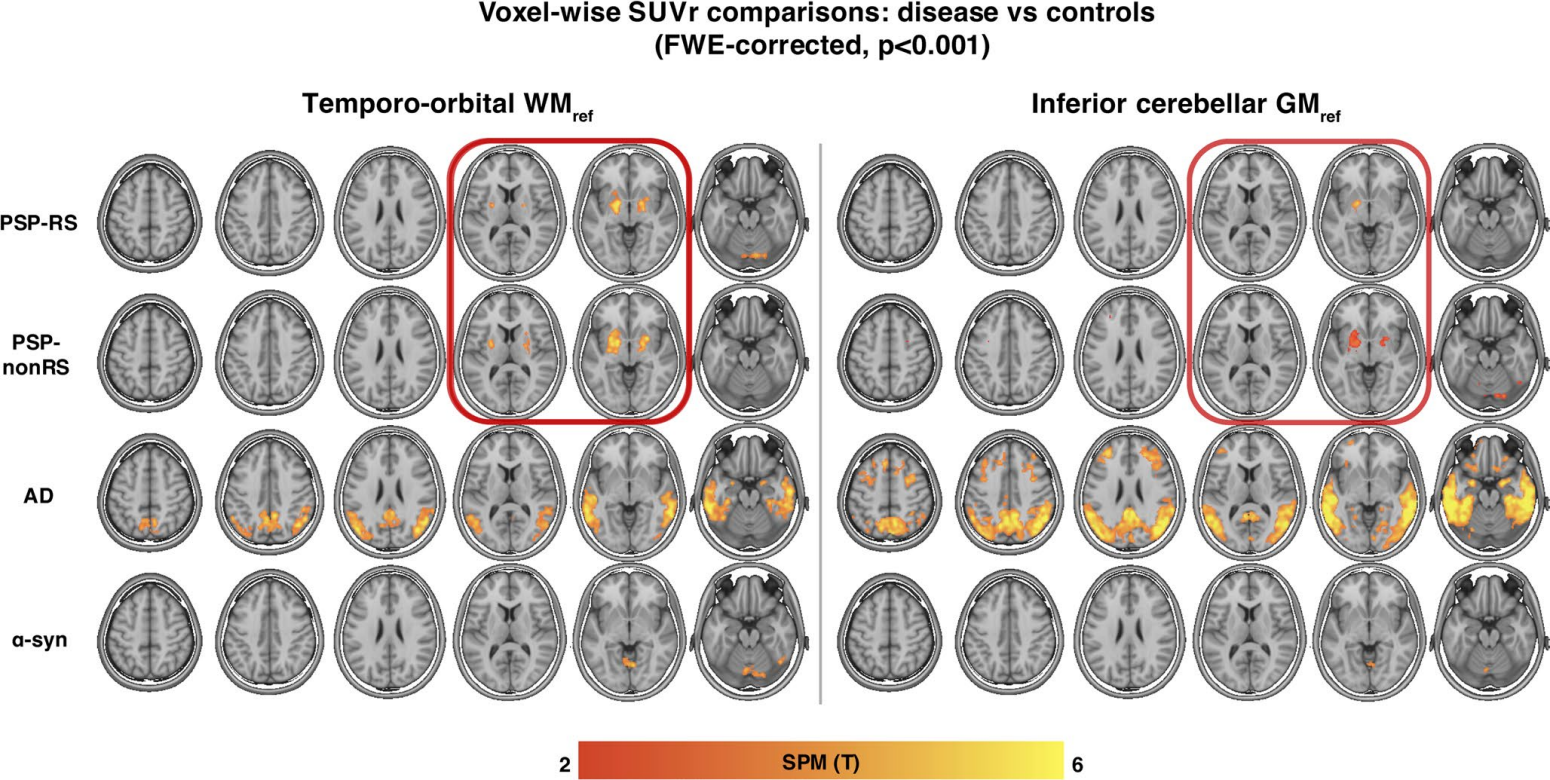
Voxel-wise Group Comparison between Disease Samples (PSP-RS, PSP-nonRS, AD, α-syn) and HC. Comparisons were conducted by utilizing 20–40 minute [^18^F]PI-2620 SUVr PET intensity-normalized with temporo-orbital WM (WM_ref_) or conventional inferior cerebellar GM (GM_ref_). Models were adjusted for sex and age and P values corrected via cluster correction (voxel *p *< 0.001, FWE-cluster correction, *p* < 0.05). Yellow voxels indicate stronger group differences

When applying the data-driven temporo-orbital WM reference to the PSP-RS sample and the independent PSP-nonRS cohort (*n* = 54) for intensity normalization, voxel-wise comparisons (*p* < 0.001, FWE-cluster corrected at *p* < 0.05) yielded strong group differences in 20–40 min SUVrs for PSP–RS vs. HC and similarly for PSP-nonRS vs. HC (Fig. 3). When applying the temporo-orbital WM reference to the disease controls, we detected cortical temporo-parietal SUVr increases in AD vs. HC, while only minor group differences were detected for α-syn vs. HC groups in meningeal regions, confirming the specificity of the temporo-orbital WM reference region as per tau positive (i.e. AD) and negative (i.e., α-syn) disease controls. To contrast these analyses against conventional PET quantification approaches, the widely used inferior cerebellar GM reference was used to calculate SUVr with the same 20–40-minute [^18^F]PI-2620 PET data for all samples. Using voxel-wise comparisons (voxel-level *p* < 0.001, FWE-cluster corrected at *p* < 0.05), only minimal group differences in the basal ganglia SUVr values were detected for PSP–nonRS vs. HC and PSP-RS vs. HC (Fig. 3). Usage of VTr’s instead of static SUVr’s yielded overall congruent results (Supp. Figure 3).

**Fig. 4:**
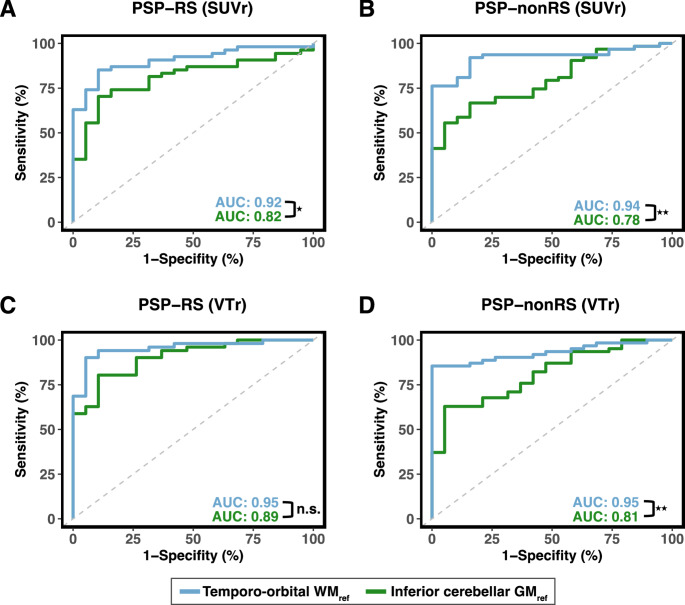
Definition of [^18^F]PI-2620 PET Cut-offs for Identifying PSP Patients**.** ROC curves were compared in their discriminative power between [^18^F]PI-2620 SUVr data (A, B) scaled either with temporo-orbital WM (blue line) or inferior cerebellar GM (green line) as reference for PSP-RS vs. HC (**A**,** C**) and PSP-nonRS vs. HC (**B**,** D**). SUVr and VTr cut-off calculation was performed and computed for both PSP samples. ROC curves were compared using a non-parametric approach as previously established by DeLong and colleagues [47]. Asterisks are used to illustrate level of significance *** = *p* < 0.001, ** = *p* < 0.01, * = *p* < 0.05. Results are reported in the text

We further ran ROC analyses to determine AUCs and [^18^F]PI-2620 PET cut-offs for separating PSP-RS and PSP-nonRS from HC groups using the PET SUVr’s or VTr’s of significant clusters in the basal ganglia displayed in Fig. 3. AUCs were compared between SUVr or VTr data scaled to either the temporo-orbital WM or the inferior cerebellar GM reference. For SUVr data within the basal ganglia clusters we found an AUC of 0.92 for PSP-RS vs. HC when using the temporo-orbital WM reference, which was significantly (*p* < 0.015) better than the AUC of 0.82 obtained when using the inferior cerebellar GM reference (Fig. 4A**)**. Congruent results were obtained for PSP-nonRS vs. HC, with AUCs of 0.94 vs. 0.78 (*p* < 0.0003) Fig. 4B) for temporo-orbital WM referenced vs. inferior cerebellar GM referenced SUVr’s or when using VTr’s instead of SUVr’s (Fig. 4C, D). Sensitivities and specificities as well as ROC-based cut-point for PSP vs. HC stratified by reference region are shown in Table 2. As sensitivity analysis, we recomputed the ROC analysis using atlas-based SUVr and VTr pallidum PET values and found congruent results (Supp. Figure 4). Overall, these findings suggest that the data-driven temporo-orbital WM reference improves the potential of [^18^F]PI-2620 PET to assess 4R tau pathology in PSP patients.

**Table 2:** Sensitivity, specificity and cut-offs for pallidum [^18^F]PI-2620 PET for PSP vs. HC comparisons

Cohort	Parameter	Reference	Sensitivity	Specificity	AUC	Cut-Off
PSP-RS	SUVr	Temporo-orbital WM	0.86	0.89	0.92	1.07
	SUVr	Inferior Cerebellum GM	0.76	0.83	0.82	1.44
	VTr	Temporo-orbital WM	0.85	0.94	0.95	1.18
	*VTr*	Inferior Cerebellum GM	0.78	0.89	0.89	1.07
PSP-nonRS	SUVr	Temporo-orbital WM	0.94	0.83	0.94	1.09
	SUVr	Inferior Cerebellum GM	0.69	0.83	0.78	1.40
	VTr	Temporo-orbital WM	0.91	0.94	0.95	1.17
	VTr	Inferior Cerebellum GM	0.64	0.94	0.81	1.23

### Targeted assessment of [^18^F]PI-2620 for quantification of 4R pallidum Tau and AD-type cortical Tau deposits

**Fig. 5:**
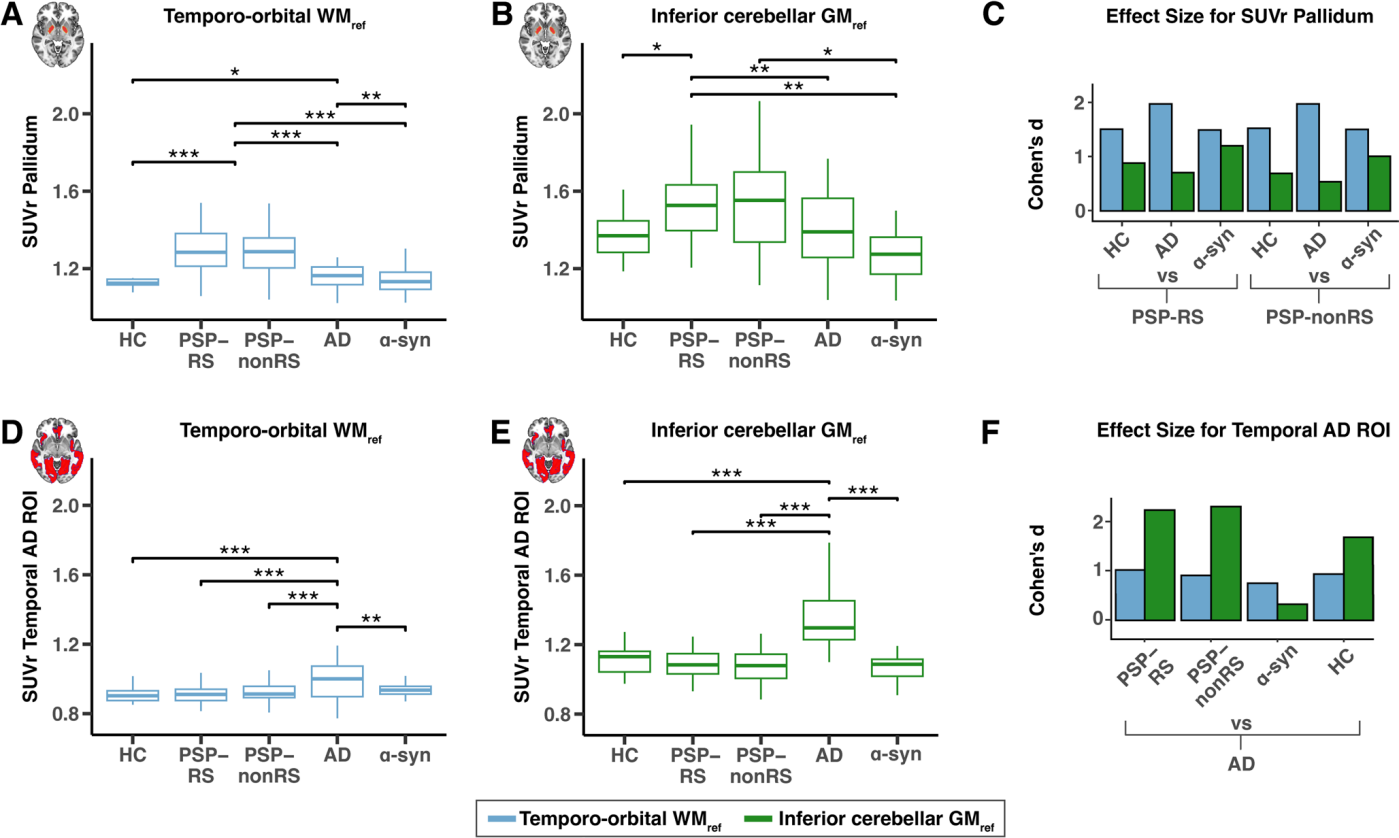
Results of ANCOVA computed for average SUVr pallidum values (**A**, **B**) and average SUVr values derived from a temporal AD signature ROI (**D**, **E**). Boxplots illustrating mean 20–40 min [^18^F]PI-2620 PET SUVr data for each cohort. SUVr data were either intensity-normalized via a temporo-orbital WM_ref_ (**A**, **D**) or with conventional inferior GM_ref_ (**B**, **E**). Barplots illustrate effect size with Cohen’s d for PSP cohorts vs. (**C**) and AD vs. control cohorts (**F**). Asterisks are used to illustrate level of significance *** = *p* < 0.001, ** = *p* < 0.01, * = *p* < 0.05. Blue: Temporo-orbital WM. Green: Inferior cerebellar GM

In a next step, we performed a targeted ROI-based analysis of [^18^F]PI-2620 to further illustrate [^18^F]PI-2620 PET signal differences between PSP vs. HC and disease controls, when specifically assessing the pallidum as a key 4R target region. In addition, we also included the temporal meta ROI as a AD-typical target region for 3/4R tau [48]. Pallidum and a temporal meta ROI SUVrs were compared across all disease samples and HC using either the temporo-orbital WM reference or the inferior cerebellar GM reference. Group comparisons of SUVr normalized with temporo-orbital WM revealed significantly elevated [^18^F]PI-2620 binding in the pallidum in patients with PSP-RS and PSP-nonRS compared to HC and disease controls (α-syn and AD) (Fig. 5A). In addition, AD patients showed slightly elevated yet significant binding differences in the globus pallidus compared to HC and α-syn. When using the conventional inferior cerebellar GM reference, we found lower, yet significant group differences in pallidum SUVr’s for PSP-RS vs. HC and disease controls, between PSP-nonRS and α-syn patients (Fig. 5B). However, there was no group difference between PSP-nonRS vs. HC and AD patients. Standardized effect sizes of these group comparisons are shown in Fig. 5C; Table 3, illustrating overall large group differences in PSP patients vs. HC and disease controls when using the temporo-orbital WM reference instead of the inferior cerebellar GM reference.

**Table 3:** Effect sizes of [^18^F]PI-2620 PET pallidum SUVr differences in PSP vs. HC and disease controls stratified by reference region

Disease group	Group comparison	Temporo-orbital WM	Inferior Cerebellum GM
PSP-RS	Cohen’s d (vs. HC)	1.48	0.68
	Cohen’s d (vs. AD)	1.94	0.53
	Cohen’s d (vs. α-syn)	1.47	0.99
PSP-nonRS	Cohen’s d (vs. HC)	0.87	1.50
	Cohen’s d (vs. AD)	0.70	1.94
	Cohen’s d (vs. α-syn)	1.18	1.48

For a targeted readout of AD-type 3/4R tau, we ran the same analyses using the AD-tailored temporal meta ROI, showing consistent group differences between AD patients and PSP, HC and α-syn patients when using the temporo-orbital WM reference (Fig. 5D) or the inferior cerebellar GM reference (Fig. 5E), yet standardized group differences were overall larger for AD patients vs. HC and disease controls when using the inferior cerebellar GM reference (Fig. 5F). Together, these results suggest that the data-driven temporo-orbital WM reference provides superior performance for the assessment of PSP, while the conventional inferior cerebellar GM reference provides superior performance for the assessment of AD. Group comparisons of VTr values revealed similar results, with elevated [^18^F]PI-2620 binding in the globus pallidus observed in patients with PSP-RS and PSP–nonRS compared to HC and disease controls (Supp. Figure 5).

### [^18^F]PI-2620 PET referenced to temporo-orbital WM reference is associated with clinical PSP severity

**Fig. 6:**
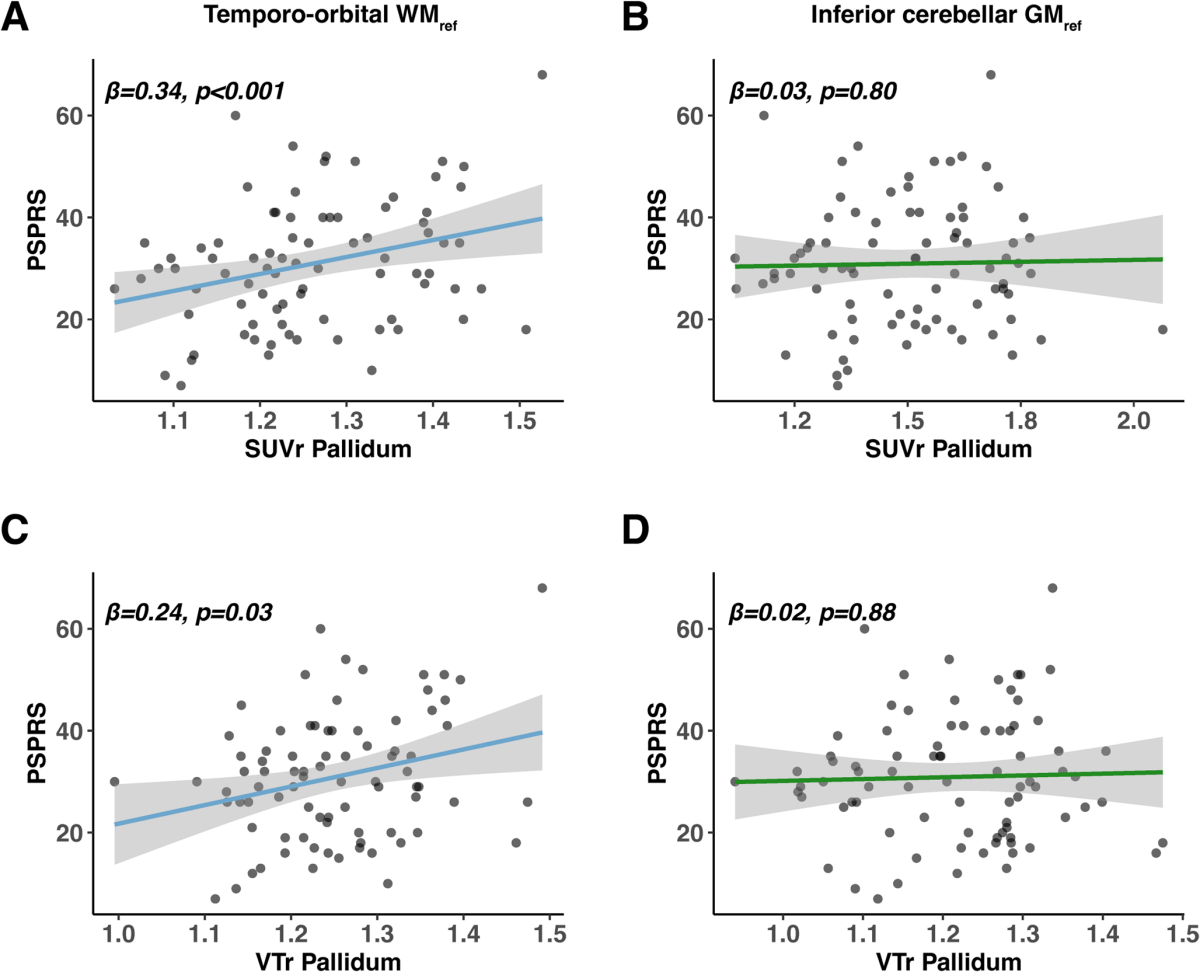
Results of Linear Regression Analyses. Scatterplots illustrating the association between mean 20–40 min [^18^F]PI-2620 PET SUVr and VTr data from the pallidum with disease severity (PSP rating scale). SUVr and VTr images were either referenced with temporo-orbital WM (**A**, **C**) or conventional inferior cerebellar GM (**B**, **D**)

Lastly, we determined whether [^18^F]PI-2620 PET quantification using the novel data-driven temporo-orbital WM allows tracking clinical disease severity. To this end, we determined the association between pallidum PET signal as a key 4R target regions and the PSPRS score, using linear regression controlling for age and sex. Since PSPRS scores were only available for a subset (i.e., PSP-RS, *n* = 40; PSP-nonRS, *n* = 40), we merged both PSP-RS and PSP-nonRS groups for this analysis to maximize statistical power. We found significant associations between higher pallidum [^18^F]PI-2620 PET and higher PSPRS scores when using SUVr (β = 0.34, *p* < 0.001, Fig. 6A) scaled to the temporo-orbital WM. In contrast, no such association was found when scaling SUVr’s to the inferior cerebellar GM reference (β = 0.03, *p* = 0.8, Fig. 6B). Congruent results were obtained for VTr’s, showing an association with PSPRS scores when scaled to the temporo-orbital WM (β = 0.24, *p* = 0.03, Fig. 6C) vs. the inferior cerebellar GM (β = 0.02, *p* = 0.88, Fig. 6D). Results were congruent when tested separately for PSP-RS and PSP-nonRS cohorts (Supp. Figs. 6 and 7). Therefore, these results suggest that higher [^18^F]PI-2620 PET signal in the pallidum as a key 4R target region is related to stronger clinical impairment, when using the novel data-driven temporo-orbital WM reference.

## Discussion

The main aim of this study was to optimize [^18^F]PI-2620 assessment as a 4R tau biomarker in PSP by addressing key limitations of the commonly used inferior cerebellar GM tau-PET reference [49], which is potentially confounded by adjacent cerebellar 4R tau accumulation [24, 33]. To address this, we obtained 60 min full dynamic [^18^F]PI-2620 PET data from PSP-RS patients (*n* = 58) and HC (*n* = 18), and computed IDIF-based VT images via referencing to carotid blood PET timeseries as a proxy of arterial tracer concentrations that are unbiased by 4R tau and corrected for differences in blood flow [36]. Using these VT images, we employed a data-driven approach to identify an optimized [^18^F]PI-2620 reference region for PSP assessment. Specifically, we performed iterative PET-intensity normalization of IDIF-derived VT images across atlas-based bilateral WM regions, based on which we benchmarked PSP-RS vs. HC group-separation using the pallidum as a 4R tau target region, adjusting for age and sex [15]. The best performing WM candidate regions surviving multiple comparison correction were then combined into a single temporo-orbital WM reference, which maximized PSP-RS vs. HC group differences in the pallidum. This data-driven temporo-orbital WM reference was then used for [^18^F]PI-2620 PET quantification in an independent validation cohort of PSP-nonRS patients (*n* = 54) and non-4R disease controls including AD (*n* = 22) and α-synucleinopathies (*n* = 21). Compared to using the conventional inferior cerebellar GM tau-PET reference [49], we found that referencing [^18^F]PI-2620 to the novel temporo-orbital WM reference yielded (i) stronger bilateral basal ganglia tau-PET signal in PSP vs. HC, (ii) larger group differences between PSP vs. disease controls in 4R target regions, and (iii) significant associations of pallidum [^18^F]PI-2620 PET signal with clinical disease severity. All results were consistent for intensity normalized VTr’s as well as static 20–40 min SUVr’s, suggesting that the novel temporo-orbital WM reference can be applied to static [^18^F]PI-2620 in PSP, thereby facilitating its’ clinical implementation via shorter 4R-tailored 20–40 min static imaging protocols [37, 38]. Together, our results suggest that a data-driven temporo-orbital WM reference for intensity scaling of [^18^F]PI-2620 PET outperforms the conventional inferior cerebellar GM reference for assessing 4R tau in PSP.

A major strength of our study is the use of non-invasively derived, reference-region-free, IDIF-based VT images for quantification of [^18^F]PI-2620 tau-PET [36]. Unlike other non-invasive PET quantification methods, this approach does not require the a priori selection of a reference tissue, which may potentially harbor 4R tau and thus bias the subsequent identification of an optimized reference region for PSP [50]. Importantly, our pre-established approach for assessing of carotid [^18^F]PI-2620 timeseries is fully automated, which further minimizes manual user input and bias [36]. We have previously shown that our fully-automated assessment of carotid [^18^F]PI-2620 PET timeseries aligns closely with invasive arterial input functions in PSP, thereby supporting its validity to derive robust and unbiased estimates of cerebral [^18^F]PI-2620 PET binding [36]. As candidate reference regions, we focused particularly on WM regions due to the following rationale: We found previously using in vivo PET imaging and post-mortem autoradiography, that [^18^F]PI-2620 captures neuronal 4R tau in GM regions, as well as oligodendroglial tau at the GM/WM boundary [25]. However, we also reported that [^18^F]PI-2620 binding and AT8 stained tau pathology drops significantly when moving away from the GM/WM boundary towards the deeper WM [25]. Therefore, we specifically eroded the atlas-based WM candidate regions, to ensure sufficient distance of WM regions from the GM/WM boundary to avoid including tau harboring tissue in our reference. In an alternative approach, a previous study involving members of our study group employed [^18^F]PI-2620 intensity normalization using the fusiform gyrus GM and cerebellar crus as a reference, yielding improved PSP vs. HC separation [51]. Of note, the fusiform gyrus is right adjacent to our suggested temporo-orbital WM reference, suggesting that the temporal lobe may in general be suitable as a 4R reference region and harbor little 4R tau as partly supported by previous post-mortem evidence in PSP [24]. However, we also argue that using cortical GM tissue as a reference may be potentially biased in PSP cases with concomitant 3/4R AD-type tau pathology, which typically targets the fusiform gyrus in early disease stages [52, 53]. This is particularly relevant, since we found previously that AD co-pathology can be found in up to 84% of patients, with ~ 10% of patients showing Braak stage III tau pathology [54], which includes the fusiform gyrus [31, 54]. Thus, we propose that the temporo-orbital WM reference may more robust than a fusiform GM reference for [^18^F]PI-2620 assessment in PSP patients with and without AD co-pathology. However, this remains to be specifically tested in PSP cases with biomarker confirmed AD co-pathology.

Overall, our results on group differences in PSP patients vs. HC align well with previous PET and autopsy studies showing that the basal ganglia resemble a key 4R tau accumulation site that are likely reflected in [^18^F]PI-2620 signal increases in PSP patients [15, 44]. Supporting the validity of [^18^F]PI-2620 to bind to 4R tau, we have shown consistently that [^18^F]PI-2620 binds to 4R tau in vitro [20], that [^18^F]PI-2620 autoradiography colocalizes with AT8 tau stainings in post-mortem PSP tissue [15, 20], and that the ante-mortem [^18^F]PI-2620 signal in the pallidum is associated with post-mortem assessed AT8 tau staining intensity and [^18^F]PI-2620 autoradiography [25]. Of note, the current study emphasizes that [^18^F]PI-2620 signal increases in PSP vs. HC become larger when using the data-driven temporo-orbital WM reference compared to the conventional cerebellar reference [15, 20, 28, 55] that may be confounded by 4R tau pathology [15, 20, 24, 28, 55]. Importantly, these results generalized fully to an independent PSP-nonRS cohort which was not involved in the data-driven identification of the temporo-orbital WM reference. Our ROC analyses further supported the superiority of the temporo-orbital WM vs. inferior cerebellar GM reference, showing increased AUCs for detecting PSP patients vs. controls, providing preliminary VTr and SUVr cut-offs for determining [^18^F]PI-2620 abnormality in clinical and research settings. Of note, the AUC increases were more pronounced in PSP-nonRS phenotypes, in some of which the cerebellum has been suggested to accumulate 4R tau earlier than in patients with a PSP-RS phenotype [24]. This suggests that employing a temporo-orbital WM reference may improve [^18^F]PI-2620-based 4R tau assessments across different clinical PSP phenotypes. Yet, this remains to be tested systematically in future studies including larger numbers of different PSP-RS and PSP-nonRS phenotypes.

When further comparing [^18^F]PI-2620 PET signal intensities between PSP patients and disease controls, we found that usage of the temporo-orbital WM reference clearly improved group separation of PSP-RS and -nonRS groups vs. disease controls compared to using the conventional inferior cerebellar reference. These findings align with previous studies reporting only weak group differences between 4R tauopathies and disease controls in typical PSP regions such as the basal ganglia when using conventional cerebellar referencing approaches [15, 20, 28, 56, 57]. We argue, that this overall stronger [^18^F]PI-2620 PET signal increase in PSP vs. HC and disease controls using the temporo-orbital reference may stem from minimizing the influence of cerebellar 4R tau which may compromise 4R tau assessments when adjusting to the cerebellum, especially in advanced PSP cases [24]. Importantly, however, though the pallidum resembles a key 4R tau aggregation site [15, 20], a recent post-mortem study has shown that 65% of AD patients show AT8 positive tau deposits the basal ganglia, which may compromise PSP vs. AD comparisons [58]. Supporting this, we also observed significant basal ganglia tau-PET signal elevations in AD vs. HC and α-syn patients using the temporo-orbital WM reference. However, PSP patients showed much stronger [^18^F]PI-2620 PET increases in the pallidum, while AD exhibited greater cortical signal elevations (i.e., in the temporal meta ROI) than PSP groups. This supports our previously suggested workflows, that simultaneous assessment of both cortical and basal ganglia [^18^F]PI-2620 PET can aid in the biomarker-based differentiation of PSP vs. AD [57]. In addition, [^18^F]PI-2620 PET signals in tau vulnerable regions in AD patients are generally higher than in patients with 4R tauopathies which can be attributed to a stronger binding of [^18^F]PI-2620 to AD-type 3/4R tau and a faster clearance for [^18^F]PI-2620 tracer in 4R tauopathies [23]. Therefore, we have previously specifically suggested 20–40 min imaging windows for assessing PSP, which capture the early and transient increases of [^18^F]PI-2620 binding to 4R tau in PSP [38]. Together, our results suggest that our novel reference region can improve the detection of PSP patients vs. HC and disease controls with overlapping clinical phenotypes (i.e. α-syn) using both dynamic scanning as well as shortened clinically feasible 20–40 min static imaging windows, thereby supporting its use as a biomarker for 4R tauopathies in clinical and research settings.

Importantly, we also detected a significant association between [^18^F]PI-2620 pallidum PET signal with clinical disease severity (i.e., PSP-RS) exclusively when using the temporo-orbital WM as a reference. This is a clear and major step forward for the clinical utility of [^18^F]PI-2620 in PSP, since we have previously failed to establish a clear and consistent association between [^18^F]PI-2620 PET and clinical disease severity [15, 55, 59]. In view of the current findings, we argue that a main confounder for assessing a link between PET signal intensity and clinical disease severity may lie in the choice of reference region. As mentioned above, cerebellar 4R tau accumulates predominantly in advanced PSP disease stages, thereby lowering the ratio of basal ganglia to inferior cerebellum PET signal in patients with more advanced clinical disease severity [24]. Hence, intensity normalization to a 4R tau harboring cerebellar reference can reduce PET signals in a 4R tau target site to a stronger degree in advanced PSP cases or rare PSP phenotypes with predominant cerebellar 4R accumulation [24]. Therefore, artificial lowering [^18^F]PI-2620 PET by a confounded reference in clinically advanced cases may mask a linear association of [^18^F]PI-2620 PET-assessed 4R tau levels with clinical scores across the PSP spectrum. This is also problematic for longitudinal studies, as it may clearly limit the opportunity to track 4R tau progression in cases where 4R tau accumulates in the cerebellum during follow up [24]. Here, our novel temporo-orbital reference may improve the detection of longitudinal [^18^F]PI-2620 PET increases, by referencing to a potentially pathology free region. We will specifically address this question in longitudinal studies, once sufficient data become available. Together, our results show that [^18^F]PI-2620 can track clinical disease severity in PSP and may therefore be suitable as a surrogate biomarker in disease modifying treatment.

When interpreting the results of our study, several limitations should be considered. First, for our data-driven approach we used a discovery cohort of PSP-RS patients and validated our results in an independent validation cohort of PSP-nonRS patients. This might be problematic as regional tau distribution may drive clinical heterogeneity in PSP, which is not detected in the current group-level analyses [24]. However, results of our voxel-wise comparisons suggested that both PSP-RS and PSP-nonRS consistently show abnormal [^18^F]PI-2620 PET levels in the basal ganglia when compared to HC and disease controls. The strong increase in sensitivity for PSP-nonRS further establishes the validity of the pallidum as a key 4R readout for both PSP-RS and nonRS phenotypes. Second, due to missing structural MRI data, we did not perform partial volume correction which might lead to spill-over effects from grey matter regions [45]. This may be particularly relevant for applying our temporo-orbital WM reference in AD cases, where we saw less pronounced group separation between AD vs. HC, suggesting that spill in of cortical temporal lobe tau-PET signal to the temporo-orbital reference. Thus, in clinical settings, we would clearly recommend using inferior cerebellar GM and temporo-orbital WM references simultenously for case-to-case assessments. We tried to address potential partial volume effects and spill-over from the GM to WM regions by eroding all candidate WM reference ROIs by a 2 mm gaussian kernel to move away from grey matter. Yet, future studies should assess whether partial volume correction can further improve [^18^F]PI-2620 PET quantification in PSP and AD. Third, PSP was diagnosed purely using clinical criteria without autopsy confirmation, hence misdiagnoses may confound our overall results. Therefore, we are currently collecting autopsy data from patients with an ante-mortem [^18^F]PI-2620 PET scan, to further investigate the sensitivity and specificity of [^18^F]PI-2620 PET for assessing 4R tau using a gold-standard post-mortem diagnosis of 4R tau. Fourth, we used an IDIF-based method to compute VT images which is only an estimate of arterial sampling [36]. While an arterial-input function based approach might be more reliable, we could previously show that IDIF-based VTs are highly correlated with arterial-input-function-based VTs and thus eligible for quantification of [^18^F]PI-2620 PET [36].

In conclusion, our findings show that [^18^F]PI-2620 PET data scaled with temporo-orbital WM is superior to conventional reference region methods for quantification of tau in 4R tauopathies and could be used in clinical trials to track disease progression or aid in molecularly defined diagnosis of PSP. We argue, these results do not only indicate a greater clinical utility of temporo-orbital reference regions in applications for differential diagnosis of patients with suspected 4R tauopathies but may also facilitate the discovery of novel PSP biomarkers (e.g. fluid) using [^18^F]PI-2620 PET imaging as an established 4R tau quantification method. Our results should act as a starting point for additional studies to focus on autopsy validation and longitudinal imaging to confirm and extend our findings and establish tau-PET imaging for PSP in the clinical setting.

## Electronic supplementary material

Below is the link to the electronic supplementary material.

Supplementary Material 1

## Data Availability

All data are available from the corresponding author upon reasonable request and upon successful conclusion of a data transfer agreement with the principal investigators at the LMU hospital. The temporo-orbital WM reference can be found on GitHub (https://github.com/Franzmeierlab).

## Abbreviations

PSP-RS: Progressive supranuclear palsy with Richardson syndrome
PSP-nonRS: Progressive supranuclear palsy with other phenotype than Richardson syndrome
IDIF: Image-Derived Input Function
SUVr: Standardized Uptake Value Ratio
VTr: Total Volume Of Distribution Ratio
ROC: Receiver operating curve
ROI: Region Of Interest
GM: Grey Matter
WM: White Matter

## References

[1] Dickson DW, et al. Office of rare diseases neuropathologic criteria for corticobasal degeneration. J Neuropathol Exp Neurol. 2002;61(11):935–46. 12430710 10.1093/jnen/61.11.935

[2] Flament S, et al. Abnormal Tau proteins in progressive supranuclear palsy. Similarities and differences with the neurofibrillary degeneration of the alzheimer type. Acta Neuropathol. 1991;81(6):591–6. 1831952 10.1007/BF00296367

[3] Dickson DW. Neuropathologic differentiation of progressive supranuclear palsy and corticobasal degeneration. J Neurol. 1999;246( Suppl 2):II6-15. 10.1007/BF03161076 10525997

[4] Rosler TW, et al. Four-repeat tauopathies. Prog Neurobiol. 2019;180:101644. 31238088 10.1016/j.pneurobio.2019.101644

[5] Graff-Radford J, et al. New insights into atypical Alzheimer’s disease in the era of biomarkers. Lancet Neurol. 2021;20(3):222–34. 33609479 10.1016/S1474-4422(20)30440-3 PMC8056394

[6] Necpal J, Borsek M, Jelenova B. Parkinson’s disease on the way to progressive supranuclear palsy: a review on PSP-parkinsonism. Neurol Sci. 2021;42(12):4927–36. 34532773 10.1007/s10072-021-05601-8

[7] Hoglinger GU, et al. Clinical diagnosis of progressive supranuclear palsy: the movement disorder society criteria. Mov Disord. 2017;32(6):853–64. 28467028 10.1002/mds.26987 PMC5516529

[8] Constantinides VC, et al. Corticobasal degeneration and corticobasal syndrome: a review. Clin Park Relat Disord. 2019;1:66–71. 34316603 10.1016/j.prdoa.2019.08.005 PMC8288513

[9] van Eimeren T, et al. Neuroimaging biomarkers for clinical trials in atypical parkinsonian disorders: proposal for a neuroimaging biomarker utility system. Alzheimers Dement (Amst). 2019;11:301–9. 30984816 10.1016/j.dadm.2019.01.011 PMC6446052

[10] Whitwell JL, et al. Radiological biomarkers for diagnosis in PSP: where are we and where do we need to be? Mov Disord. 2017;32(7):955–71. 28500751 10.1002/mds.27038 PMC5511762

[11] Scholl M, et al. Distinct 18F-AV-1451 Tau PET retention patterns in early- and late-onset Alzheimer’s disease. Brain. 2017;140(9):2286–94. 29050382 10.1093/brain/awx171

[12] Ossenkoppele R, et al. Tau PET patterns mirror clinical and neuroanatomical variability in Alzheimer’s disease. Brain. 2016;139(Pt 5):1551–67. 26962052 10.1093/brain/aww027 PMC5006248

[13] Coakeley S, et al. Positron emission tomography imaging of Tau pathology in progressive supranuclear palsy. J Cereb Blood Flow Metab. 2017;37(9):3150–60. 28155586 10.1177/0271678X16683695 PMC5584690

[14] Groot C, et al. Tau PET imaging in neurodegenerative disorders. J Nucl Med. 2022;63(Suppl 1):S20–6. 10.2967/jnumed.121.263196 35649647

[15] Brendel M, et al. Assessment of 18F-PI-2620 as a biomarker in progressive supranuclear palsy. JAMA Neurol. 2020;77(11):1408–19. 33165511 10.1001/jamaneurol.2020.2526 PMC7341407

[16] Li CH, et al. Integrated (18)F-T807 Tau PET, structural MRI, and plasma Tau in tauopathy neurodegenerative disorders. Front Aging Neurosci. 2021;13:646440. 33854426 10.3389/fnagi.2021.646440 PMC8039308

[17] Kunze G, et al. Molecular simulations reveal distinct energetic and kinetic binding properties of [(18)F]PI-2620 on Tau filaments from 3R/4R and 4R tauopathies. ACS Chem Neurosci. 2022;13(14):2222–34. 35762647 10.1021/acschemneuro.2c00291

[18] Kroth H, et al. Discovery and preclinical characterization of [(18)F]PI-2620, a next-generation Tau PET tracer for the assessment of Tau pathology in Alzheimer’s disease and other Tauopathies. Eur J Nucl Med Mol Imaging. 2019;46(10):2178–89. 31264169 10.1007/s00259-019-04397-2 PMC6667408

[19] Zhou Y, et al. Dissecting the binding profile of PET tracers to corticobasal degeneration Tau fibrils. ACS Chem Neurosci. 2021;12(18):3487–96. 34464084 10.1021/acschemneuro.1c00536 PMC8447187

[20] Franzmeier N, et al. Tau deposition patterns are associated with functional connectivity in primary tauopathies. Nat Commun. 2022;13(1):1362. 35292638 10.1038/s41467-022-28896-3 PMC8924216

[21] Messerschmidt K, et al. (18)F-PI-2620 Tau PET improves the imaging diagnosis of progressive supranuclear palsy. J Nucl Med. 2022 63:1754–60 10.2967/jnumed.121.262854 PMC9635682 35422444

[22] Palleis C, et al. Cortical [(18) F]PI-2620 Binding Differentiates Corticobasal Syndrome Subtypes*.* Mov Disord. 2021. 10.1002/mds.28624 33951244

[23] Song M, et al. Binding characteristics of [(18)F]PI-2620 distinguish the clinically predicted Tau isoform in different tauopathies by PET. J Cereb Blood Flow Metab. 2021;41(11):2957–72. 34044665 10.1177/0271678X211018904 PMC8545042

[24] Kovacs GG, et al. Distribution patterns of Tau pathology in progressive supranuclear palsy. Acta Neuropathol. 2020;140(2):99–119. 32383020 10.1007/s00401-020-02158-2 PMC7360645

[25] Slemann L, et al. Neuronal and oligodendroglial, but not astroglial, Tau translates to in vivo Tau PET signals in individuals with primary tauopathies. Acta Neuropathol. 2024;148(1):70. 39580770 10.1007/s00401-024-02834-7 PMC11586312

[26] Schonecker S, et al. Symptomatology in 4-repeat tauopathies is associated with data-driven topology of [(18)F]-PI-2620 tau-PET signal. Neuroimage Clin. 2023;38:103402. 37087820 10.1016/j.nicl.2023.103402 PMC10300609

[27] Malpetti M, et al. Neuroinflammation parallels 18F-PI-2620 positron emission tomography patterns in primary 4-Repeat tauopathies. Mov Disord. 2024;39(9):1480–92. 39022835 10.1002/mds.29924

[28] Roemer SN, et al. Subcortical tau is linked to hypoperfusion in connected cortical regions in 4-repeat tauopathies. Brain. 2024;147(7):2428–39. 38842726 10.1093/brain/awae174

[29] Leuzy A, et al. Tau PET imaging in neurodegenerative tauopathies-still a challenge. Mol Psychiatry. 2019;24(8):1112–34. 30635637 10.1038/s41380-018-0342-8 PMC6756230

[30] Bollack A, et al. Longitudinal amyloid and Tau PET imaging in Alzheimer’s disease: a systematic review of methodologies and factors affecting quantification. Alzheimers Dement. 2023;19(11):5232–52. 37303269 10.1002/alz.13158

[31] Braak H, Braak E. Neuropathological stageing of Alzheimer-related changes. Acta Neuropathol. 1991;82(4):239–59. 1759558 10.1007/BF00308809

[32] Koga S, et al. Cerebellar ataxia in progressive supranuclear palsy: an autopsy study of PSP-C. Mov Disord. 2016;31(5):653–62. 26841329 10.1002/mds.26499 PMC4861661

[33] Piao YS, et al. Cerebellar cortical tau pathology in progressive supranuclear palsy and corticobasal degeneration. Acta Neuropathol. 2002;103(5):469–74. 11935262 10.1007/s00401-001-0488-2

[34] Aguero C, et al. Head-to-head comparison of [(18)F]-Flortaucipir, [(18)F]-MK-6240 and [(18)F]-PI-2620 postmortem binding across the spectrum of neurodegenerative diseases. Acta Neuropathol. 2024;147(1):25. 38280071 10.1007/s00401-023-02672-z PMC10822013

[35] Kling A, et al. Exploring the origins of frequent tau-PET signal in vermal and adjacent regions. Eur J Nucl Med Mol Imaging. 2025. 10.1007/s00259-025-07199-x. 10.1007/s00259-025-07199-x PMC12316827 40100387

[36] Meindl M, et al. Assessment of [(18)F]PI-2620 Tau-PET quantification via non-invasive automatized image derived input function. Eur J Nucl Med Mol Imaging. 2024;51(11):3252–66. 38717592 10.1007/s00259-024-06741-7 PMC11368995

[37] Bauer T, et al. Pragmatic algorithm for visual assessment of 4-Repeat tauopathies in [(18)F]PI-2620 PET scans. NeuroImage. 2025;306:121001. 39798829 10.1016/j.neuroimage.2025.121001

[38] Song M, et al. Feasibility of short imaging protocols for [(18)F]PI-2620 tau-PET in progressive supranuclear palsy. Eur J Nucl Med Mol Imaging. 2021;48(12):3872–85. 34021393 10.1007/s00259-021-05391-3 PMC8484138

[39] Armstrong MJ, et al. Criteria for the diagnosis of corticobasal degeneration. Neurology. 2013;80(5):496–503. 23359374 10.1212/WNL.0b013e31827f0fd1 PMC3590050

[40] Jack CR Jr., et al. Revised criteria for diagnosis and staging of Alzheimer’s disease: Alzheimer’s association workgroup. Alzheimers Dement. 2024;20(8):5143–69. 38934362 10.1002/alz.13859 PMC11350039

[41] Golbe LI, Ohman-Strickland PA. A clinical rating scale for progressive supranuclear palsy. Brain. 2007;130(Pt 6):1552–65. 17405767 10.1093/brain/awm032

[42] Logan J, et al. Graphical analysis of reversible radioligand binding from time-activity measurements applied to [N-11 C-methyl]-(-)-cocaine PET studies in human subjects. J Cereb Blood Flow Metab. 1990;10(5):740–7. 2384545 10.1038/jcbfm.1990.127

[43] Hammers A, et al. Three-dimensional maximum probability atlas of the human brain, with particular reference to the temporal lobe. Hum Brain Mapp. 2003;19(4):224–47. 12874777 10.1002/hbm.10123 PMC6871794

[44] Marotta C, et al. Biomarkers of disease progression in progressive supranuclear palsy for use in clinical trials. Brain Commun. 2025;7(1):fcaf022. 39882025 10.1093/braincomms/fcaf022 PMC11775610

[45] Rullmann M, et al. Partial-volume effect correction improves quantitative analysis of 18F-Florbetaben beta-Amyloid PET scans. J Nucl Med. 2016;57(2):198–203. 26541776 10.2967/jnumed.115.161893

[46] R Core Team. R: A Language and environment for statistical computing. R Foundation for Statistical Computing; 2021.

[47] DeLong ER, DeLong DM, Clarke-Pearson DL. Comparing the areas under two or more correlated receiver operating characteristic curves: a nonparametric approach. Biometrics. 1988;44(3):837–45. 3203132

[48] Jack CR Jr., et al. Longitudinal Tau PET in ageing and Alzheimer’s disease. Brain. 2018;141(5):1517–28. 29538647 10.1093/brain/awy059 PMC5917767

[49] Baker SL, Maass A, Jagust WJ. Considerations and code for partial volume correcting [(18)F]-AV-1451 Tau PET data. Data Brief. 2017;15:648–57. 29124088 10.1016/j.dib.2017.10.024 PMC5671473

[50] Volpi T, et al. An update on the use of image-derived input functions for human PET studies: new hopes or old illusions? EJNMMI Res. 2023;13(1):97. 37947880 10.1186/s13550-023-01050-w PMC10638226

[51] Bischof GN, et al. Improved Tau PET SUVR quantification in 4-Repeat Tau phenotypes with [(18)F]PI-2620. J Nucl Med. 2024;65(6):952–5. 38575191 10.2967/jnumed.123.265930 PMC11149601

[52] Schöll M, et al. PET imaging of Tau deposition in the aging human brain. Neuron. 2016;89(5):971–82. 26938442 10.1016/j.neuron.2016.01.028 PMC4779187

[53] Rullmann M, et al. Multicenter (18)F-PI-2620 PET for in vivo braak staging of tau pathology in Alzheimer’s disease. Biomolecules. 2022;12(3):458. 10.3390/biom12030458 PMC8946049 35327650

[54] Jecmenica Lukic M, et al. Copathology in progressive supranuclear palsy: does it matter?? Mov Disord. 2020;35(6):984–93. 32125724 10.1002/mds.28011

[55] Messerschmidt K, et al. (18)F-PI-2620 Tau PET improves the imaging diagnosis of progressive supranuclear palsy. J Nucl Med. 2022;63(11):1754–60. 35422444 10.2967/jnumed.121.262854 PMC9635682

[56] Blazhenets G, et al. [(18)F]PI-2620 binding patterns in patients with suspected alzheimer disease and frontotemporal lobar degeneration. J Nucl Med. 2023;64(12):1980–9. 37918868 10.2967/jnumed.123.265856 PMC10690126

[57] Dilcher R, et al. Combining cerebrospinal fluid and PI-2620 tau-PET for biomarker-based stratification of alzheimer’s disease and 4R-tauopathies. Alzheimers Dement. 2024;20(10):6896–909. 39263969 10.1002/alz.14185 PMC11485081

[58] Hamasaki H, et al. Tauopathy in basal ganglia involvement is exacerbated in a subset of patients with alzheimer’s disease: the Hisayama study. Alzheimers Dement (Amst). 2019;11:415–23. 31206007 10.1016/j.dadm.2019.04.008 PMC6558096

[59] Katzdobler S, et al. Additive value of [(18)F]PI-2620 perfusion imaging in progressive supranuclear palsy and corticobasal syndrome. Eur J Nucl Med Mol Imaging. 2023;50(2):423–34. 36102964 10.1007/s00259-022-05964-w PMC9816230

