# Supplementary material for: Developing a novel reference region for [18F]PI-2620-PET imaging to facilitate the assessment of 4-repeat tauopathies
Source: Eur J Nucl Med Mol Imaging. 2025 Jun 10;52(13):5098–112. doi: 10.1007/s00259-025-07396-8 (PMC12589379; doi:10.1007/s00259-025-07396-8)
Supplement: Supplementary file 1 — Supplementary Material 1 [file 259_2025_7396_MOESM1_ESM.docx]

**Supplementary Materials**

**Developing a Novel Reference Region for [^18^F]PI-2620-PET Imaging to Facilitate the Assessment of 4-Repeat Tauopathies**

Lukas Frontzkowski^1,2*^, Johannes Gnörich^2*^, Mattes Gross^1,2*^, Amir Dehsarvi,^1^ Sebastian N. Roemer-Cassiano^1,3^, Carla Palleis^3,4,5^, Sabrina Katzdobler^3^, Anna Dewenter^1^, Anna Steward^1^, Davina Biel^1^, Fabian Hirsch^1^, Zeyu Zhu^1^, Johannes Levin,^3,4,5^ Andrew W. Stephens^6^, Andre Müller^6^, Norman Koglin^6^, Gérard N. Bischof^6^, Gabor G. Kovacs^7,8^, Günter U. Höglinger^3,4,5^, Matthias Brendel^2,4,5#^, Nicolai Franzmeier^1,4,9#^

1. Institute for Stroke and Dementia Research (ISD), University Hospital, LMU Munich, Germany
2. Department of Nuclear Medicine, LMU University Hospital, Munich, Germany
3. Department of Neurology, LMU University Hospital, Munich, Germany
4. Munich Cluster for Systems Neurology (SyNergy), Munich, Germany
5. German Center for Neurodegenerative Diseases (DZNE), Munich, Germany
6. Life Molecular Imaging, GmbH, Berlin, Germany
7. Tanz Centre for Research in Neurodegenerative Disease (CRND), Toronto, Canada
8. Laboratory Medicine Program and Krembil Brain Institute, University Health Network, Toronto, Canada
9. University of Gothenburg, The Sahlgrenska Academy, Institute of Neuroscience and Physiology, Department of Psychiatry and Neurochemistry, Mölndal and Gothenburg, Sweden

**Supplementary Table 1**

| Hammers Atlas ROI | T | P |
| --- | --- | --- |
| Medial and inferior temporal gyrus | -6.827880 | <0.0001 |
| Anterior temporal lobe lateral part | -6.663098 | <0.0001 |
| Anterior temporal lobe medial part | -6.386017 | <0.0001 |
| Anterior orbital gyrus | -6.135988 | <0.0001 |

**Supplementary Figure 1**

**Supplementary Figure 1**. Average [^18^F]PI-2620 PET SUVr's for PSP-RS, PSP-nonRS and HC.

**
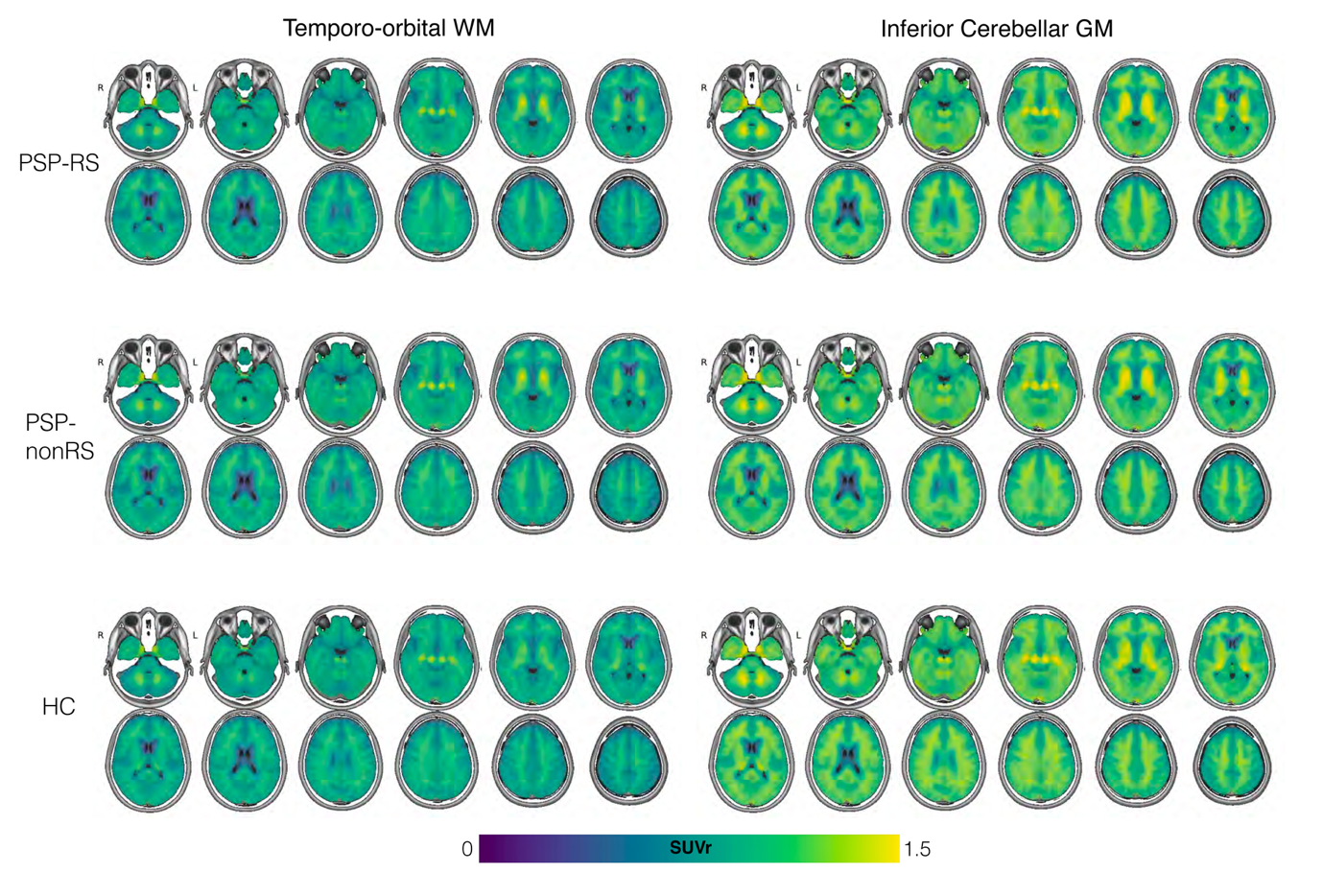
**

**Supplementary Figure 2**

**
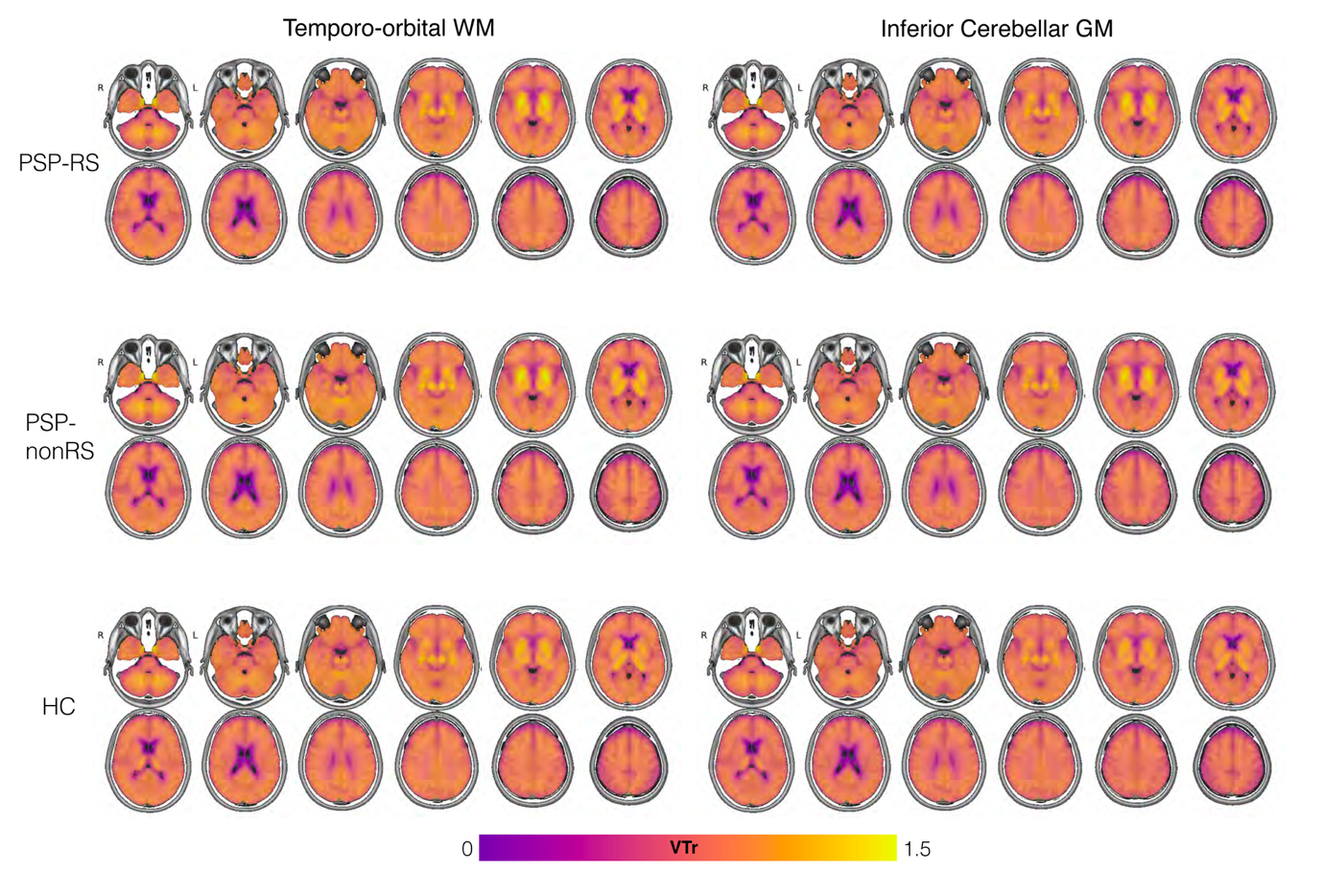
**

**Supplementary Figure 2**. Average [^18^F]PI-2620 PET VTr's for PSP-RS, PSP-nonRS and HC.

**Supplementary Figure 3**

**Supplementary Figure 3.** **Voxel-wise Group Comparisons between Disease Samples (PSP-RS, PSP-nonRS, AD, α-syn) and HC**. Comparisons were conducted by utilizing [^18^F]PI-2620 VTr PET intensity-normalized with temporo-orbital WM (WM_ref_) or conventional inferior cerebellum GM (GM_ref_). Models were adjusted for sex and age and P values corrected via cluster correction (voxel p<0.001, FWE-cluster correction, p<0.05). Yellow voxels indicate stronger group differences.

*
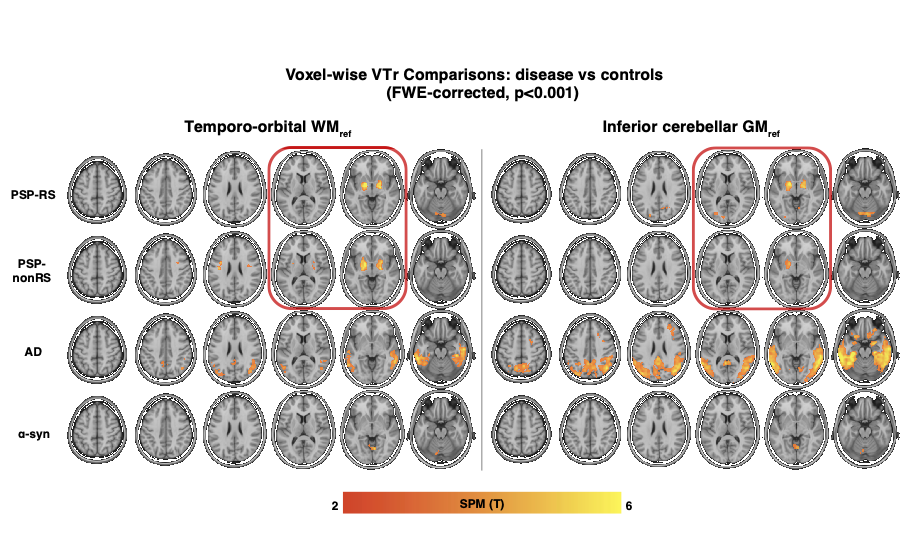
*

**Supplementary Figure 4**

**Supplementary Figure 4.** **Definition of [^18^F]PI-2620 PET Cut-offs for Identifying PSP Patients using atlas-based pallidum PET.** ROC curves were compared in their discriminative power between [^18^F]PI-2620 SUVr data (A, B) scaled either with temporo-orbital WM (blue line) or inferior cerebellar GM (green line) as reference for PSP-RS vs HC (**A,C**) and PSP-nonRS vs HC (**B,D**). SUVr and VTr cut-off calculation was performed and computed for both PSP samples. ROC curves were compared using a non-parametric approach as previously established by DeLong and colleagues. Asterisks are used to illustrate level of significance *** = p<0.001, ** = p<0.01, * = p<0.05.

**Supplementary Figure 5**
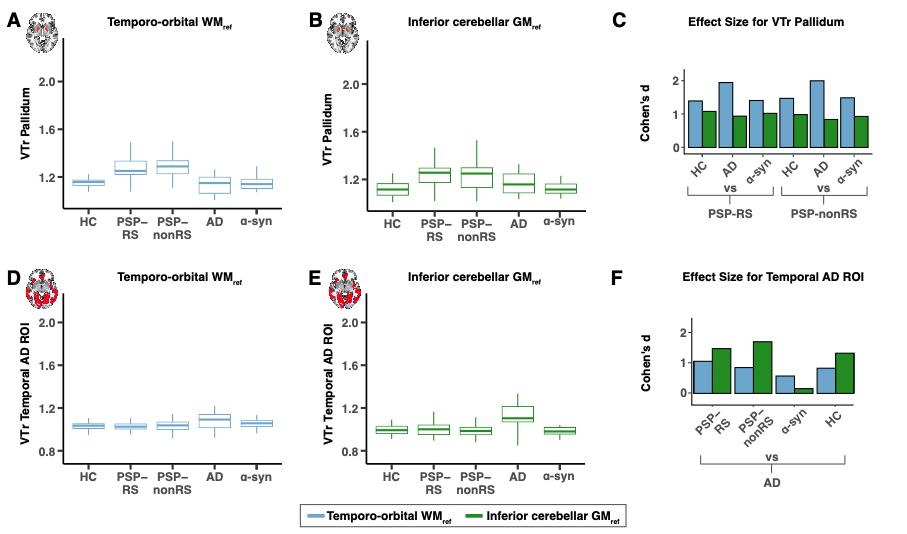


**Supplementary Figure 5.** Results of ANCOVA computed for average VTr pallidum values (**A, B**) and average VTr values derived from a temporal AD signature ROI (**D,E**). Boxplots illustrating mean [^18^F]PI-2620 PET VTr data for each cohort. VTr data were either intensity-normalized via a temporo-orbital WM reference region (**A**, **D**) or with conventional inferior cerebellar grey matter (**B**, **E**). Barplots illustrate effect size with Cohen's d for PSP cohorts vs (**C**) and AD vs control cohorts (**F**). Blue: Temporo-orbital WM. Green: Inferior Cerebellar GM.

**Supplementary Figure 6**


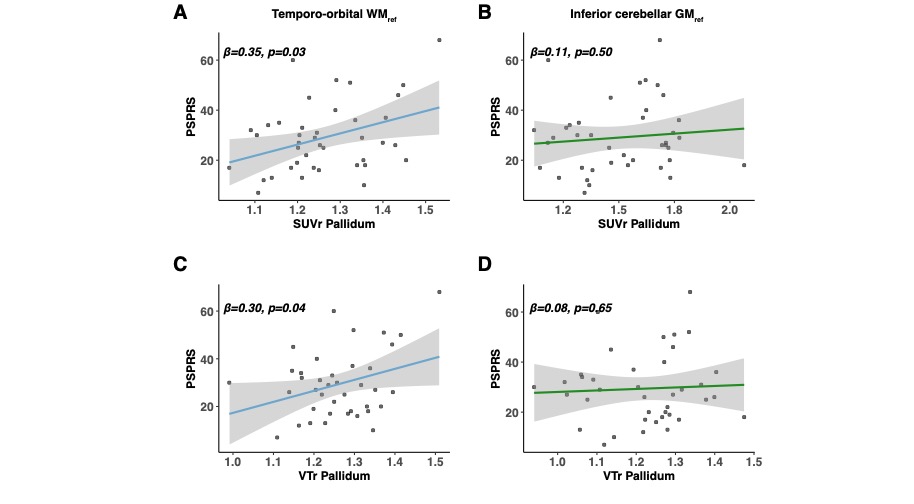


**Supplementary Figure 6 Results of Linear Regression Analyses: PSP-nonRS**. Scatterplots illustrating the association between mean 20-40 minute [^18^F]PI-2620 PET SUVr and VTr data from the pallidum with disease severity (PSP rating scale). SUVr and VTr images were either referenced with temporo-orbital WM (**A**,**C**) or conventional inferior cerebellar GM (**B**,**D**).

**Supplementary Figure 7**


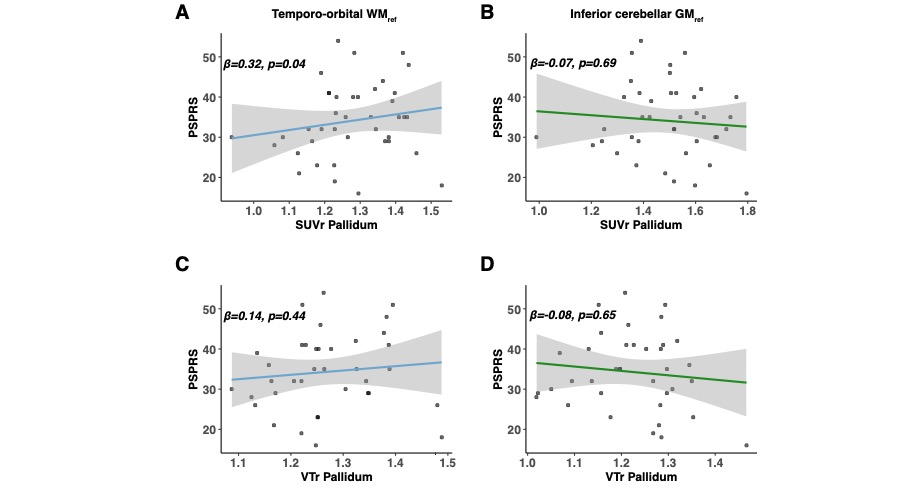


**Supplementary Figure 7 Results of Linear Regression Analyses: PSP-RS**. Scatterplots illustrating the association between mean 20-40 minute [^18^F]PI-2620 PET SUVr and VTr data from the pallidum with disease severity (PSP rating scale). SUVr and VTr images were either referenced with temporo-orbital WM (**A**,**C**) or conventional inferior cerebellar GM (**B**,**D**).
